# Challenges and Paradoxes of Human Factors in Health Technology Design

**DOI:** 10.2196/humanfactors.4653

**Published:** 2016-03-01

**Authors:** Plinio P Morita, Joseph A Cafazzo

**Affiliations:** ^1^ Healthcare Human Factors Techna Institute University Health Network Toronto, ON Canada; ^2^ Institute of Health Policy, Management, and Evaluation Dalla Lana School of Public Health University of Toronto Toronto, ON Canada; ^3^ Institute of Biomaterials and Biomedical Engineering Faculty of Applied Science and Engineering University of Toronto Toronto, ON Canada

**Keywords:** equipment design, task performance and analysis, workflow, workload

## Abstract

Usability testing allows human factors professionals to identify and mitigate issues with the design and use of medical technology. The test results, however, can be paradoxical and therefore be misinterpreted, limiting their usefulness. The paradoxical findings can lead to products that are not aligned with the needs and constraints of their users. We herein report on our observations of the paradox of expertise, the paradox of preference versus performance, and the paradox of choice. Each paradox explored is in the perspective of the design of medical technology, the issues that need to be considered in the interpretation of the test results, as well as suggestions on how to avoid the pitfalls in the design of medical technology. Because these paradoxes can influence product design at various stages of product development, it is important to be aware of the effects to interpret the findings properly.

## Introduction

Usability testing is of prime importance in evaluating technology designs. Usability testing can be a powerful tool to validate a design, while equally being useful at identifying flaws. However, when confronted with paradoxical findings, designers and engineers are often left in turmoil over the challenges of interpreting usability test results.

Usability testing as a scientific method is still subject to the limitations of being mostly based on subjective, qualitative evaluations [1]. The data collected during the testing process highly rely on the experience of the experimenter [2], how the experimenter interacts with the participants [3], and the individuals participating in the study [4]. If testing is not done according to established norms of qualitative research, the process ultimately has the potential to result in erroneous findings.

At times, the process of usability testing can also result in some surprising, contradictory, and often-paradoxical findings that may leave human factors professionals (HFPs) perplexed. Only when these paradoxical findings are explained and properly understood by the HFPs can the findings be properly interpreted and the value of the testing be derived in the iterative development process.

In the past decade, hundreds of products have been tested in the usability labs at Toronto General Hospital, part of the University Health Network. During that time, HFPs have routinely identified paradoxical findings on usability tests, which at times, could have led to misinterpretations and erroneous conclusions that in turn could have negatively affected product design.

In this paper, we will explore three paradoxes of health technology design that can confound and mislead both designers and engineers in developing health technologies. These were the most prominent paradoxes identified over the years and the three that could have the most negative impact on design if not accounted for during the evaluations.

## The Paradoxes

### The Paradox of Expertise: “Do As I Do, Not As I Say”

An iterative, user-centered design (UCD) process of health technology, analogous to any product development, focuses on the use of expert knowledge to identify the requirements, constraints, and features to be included in the final product. Subject-matter experts (nurses, physicians, allied health professionals, among other care providers and patients) are involved in the early stages of product development through interviews and focus groups. Their feedback forms the basis of system’s specifications [5-7]. These experts are integrated into the design process because they are considered to bring domain knowledge that is otherwise not available to the design team.

Consequently, as experts, their interactions with the medical technology under development are influenced by their extensive knowledge and well-aligned, mature mental models [8,9]. According to Rasmussen’s *Skills, Rules, and Knowledge* framework—which describes why operators with varied levels of expertise and training will behave differently and have distinct psychological processes—these interactions are not necessarily shared by all individuals. These interactions present a unique and refined view of how the expert subset of users interprets the work domain and the interaction with the system [10].

The main premise is that experts will offer greater knowledge in defining product requirements, defining workflows, etc. However, the issue with this approach is that the health technology being developed now reflects only the interactions and constraints of a small percentage of the total users (often, only expert users) who will be interacting with it. The finely tuned mental models of expert individuals are not shared by the majority of the less-experienced users, as described by Hmelo-Silver and Pfeffer [11]. Consequently, the final evaluation only provides a partial view of product specifications because these expert users can have significantly different needs from the health technology being developed. In addition, expert users may have mature mental models that can result in users using shortcuts when interacting with the medical technology and consequently missing important issues with the design. As a result, product specifications identified by experts might significantly differ from the needs of the larger majority of users of the technology.

Some aspects of user interaction design of the system might be left out as a result of the inputs from experts, as their cognitive pathways have allowed them to bypass those components of the workflow. As described by Firesmith [12], “subject matter experts who specify requirements often take certain information for granted and omit it, even though it is not obvious to other stakeholders of the requirement” (p 79). This is the essence of the *paradox of expertise*. In the same direction, the use of expert knowledge can also result in lack of innovation, as experts are usually locked in their own ways, and may demonstrate resistance to innovation.

In our own practice, it has also been observed that there is a very sharp inconsistency and incompleteness between the verbal description of the work performed by experts and how they actually perform their work in the field. This is in alignment with what was discussed by Benner [9] in a previous work where she identifies the difficulty of gathering data from experts and how these expert individuals usually use cognitive shortcuts that they are often not aware of [6,10]. Because their actions and decisions are highly rooted on skill-based behaviors [10], expert’s descriptions of the work might be simplistic as they do not fully perceive the wide range of constraints that affect their work. Ultimately, this can potentially lead to distorted representations of the work domain.

**Figure 1:**
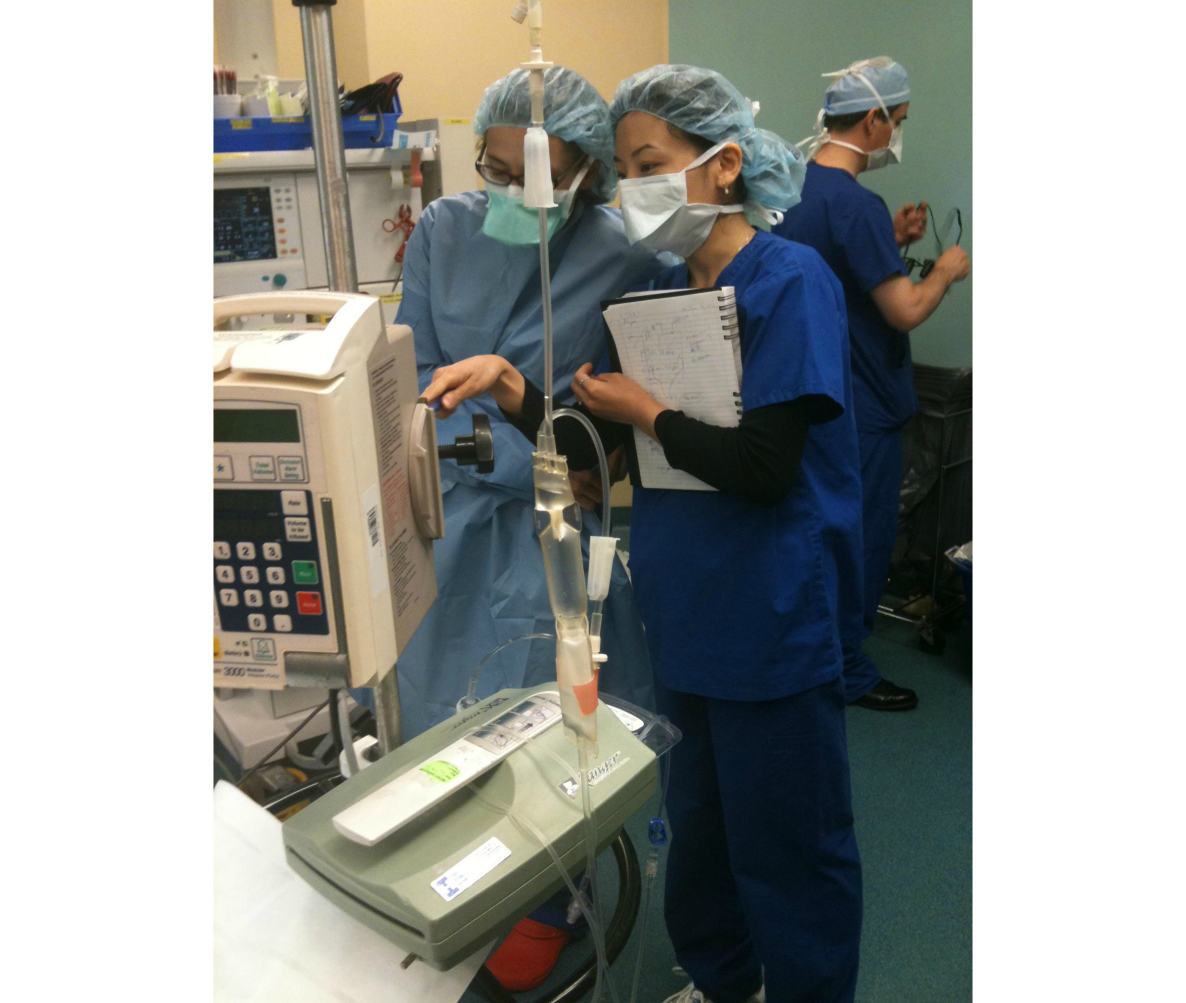
Human factors expert embedded in an operating room environment at University Health Network, gaining a deeper understanding of how clinicians actually work.

The gap between description and performance reinforces the importance of using other ethnographic tools such as in situ observations as part of the requirements gathering and design process as shown in Figure 1 [6,7]. These methods would allow designers to analyze the work domain in situ and gather data without the bias of an expert’s limitation. In the requirements gathering stage, designers must ensure that they avoid a distorted representation of the workflow, feature set, and other specifications. It would only be through direct observation that designers could fully comprehend the domain and properly incorporate constraints and requirements into the system.

When designing for complex systems, the lack of complete understanding of the domain can result in a flat information architecture design that leads to a crowded, seemingly complex user interface. Because the designer does not have complete insights into what is important to the user, the final design often lacks the necessary hierarchy of information or functionality that maps to the users’ mental model.

For example, in the radiation therapy domain [13], only through proper ethnography were the authors able to identify that the checking procedures during radiation therapy were often skipped because the task was too complex, time consuming, or distractions happened [13]. When asked, professionals would normally state that all checks had been performed. As such, it was necessary for the researchers to be present while tasks were being performed to identify that the skipping had actually happened. This demonstrates that it was only through direct observation that researchers were able to understand the real issues and identify ways to address them. This is a good example of the *paradox of expertise*, where it was important to rely on observed data rather than on verbal reports [13].

The original architecture provided users with the necessary information for the checks, but this information was spread across multiple screens without any logical structure. The authors brought forward items that were previously buried in the interface and difficult for users to locate. By reorganizing the information architecture and through forcing functions in the form of simplified automated checklists, the authors were able to significantly improve the checking process and patient safety. The new interface, when compared with the original one, showed improved error detection rates and high user satisfaction [13].

Design decisions must be made based on a combination of user-reported data and observed data to ensure that the system is designed for how users actually use it, instead of being designed to how they think they would use it. Although design requirements might be gathered through expert interviews and focus groups, only though the use of observational techniques can designers have a rich understanding of the work domain and the system’s hierarchy of information.

### The Paradox of Preference Versus Performance: How Could Someone Like Something They Cannot Use?

One would expect that when evaluating two possible designs, users would prefer the design in which they had greater success during testing. Oddly, that is not always the case, leaving the HFPs to conclude that the testing was somehow flawed, or they just disregard that user’s opinion altogether.

How could someone like something they cannot use?

Contrary to these paradoxical findings, Nielsen and Levy [14] described a positive correlation between user preference and user performance showing that, in general, users prefer systems in which they also performed the best. However, the same authors also argue that there are still many cases in which users prefer systems in which they perform worse. Although users are described to prefer situations in which preference and performance align [14,15], we have identified cases over years of product testing to consider these paradoxical findings as a risk.

As design methods have evolved, more approaches have been made available to influence user behavior by making simple changes in the aesthetics of the device or by using a seemingly novel and engaging control interface. New features might drive users to prefer a particular design simply due to increased affinity for that experience.

Powerful persuasive design can be used to guide how users perform certain tasks, influence user interaction, and drive user behavior. Similarly, design techniques can be used to capture users’ attention and persuade them to react positively to a design, which could be flawed or create negative outcomes [16]. Seemingly novel features and a more aesthetically pleasing design of health technology may drive users’ preference, but these do not necessarily result in better task performance.

**Figure 2:**
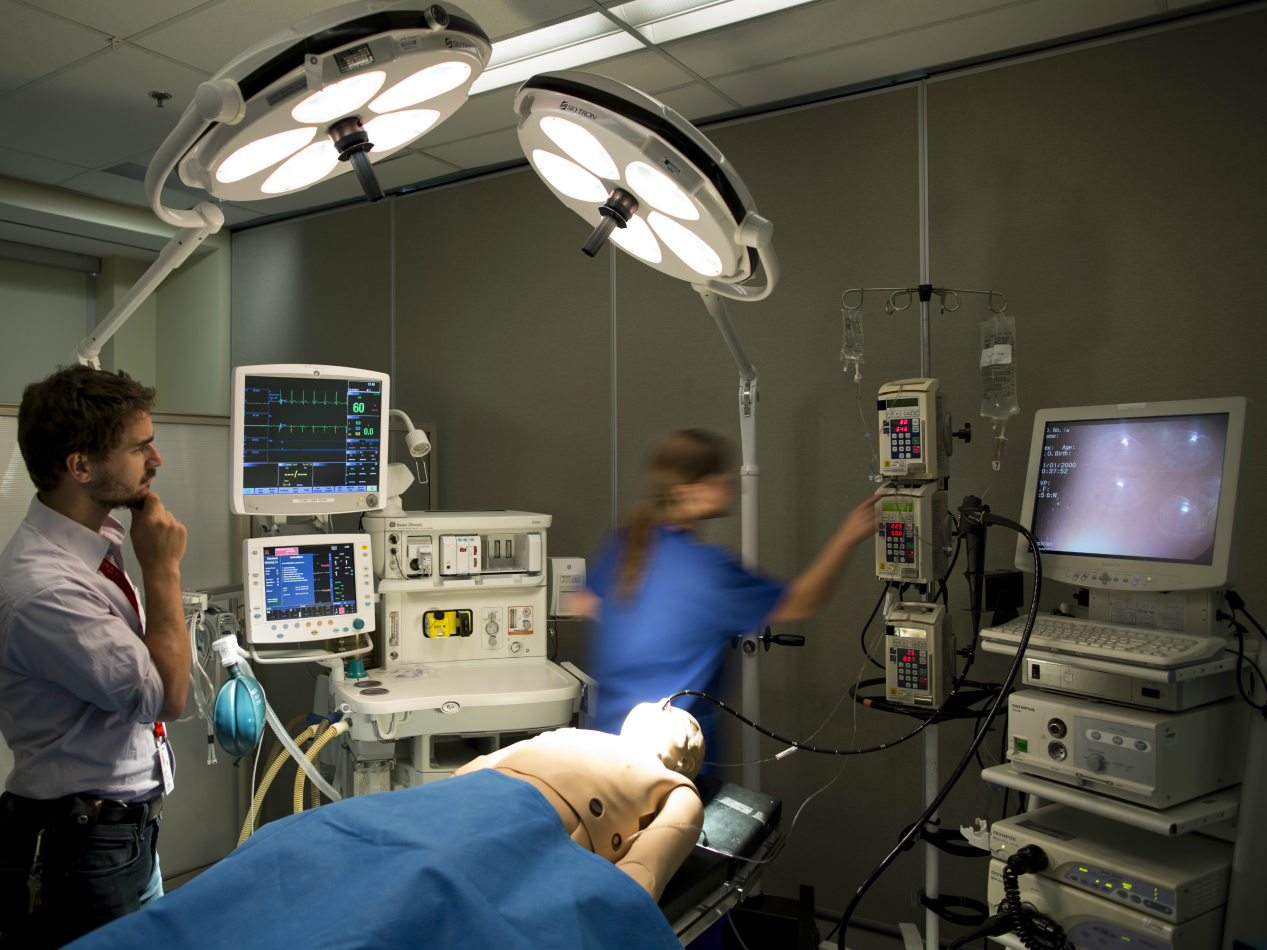
Usability labs at Toronto General Hospital showing a complete set up of a simulated operating room (including a patient simulator).

In practice at Toronto General Hospital’s usability labs (Figure 2), cases have been observed where the color palette of a device had a greater influence on nursing preference than on its usability. In this case, the observation of the paradox was further reinforced by the novel user interface of a scroll wheel that nurses found interesting and engaging to use, but did not result in successfully completing tasks. User preference, evaluated through questionnaires, demonstrated that nurses preferred the new device design. Observational and performance data, however, showed that their performance was suboptimal. Besides, the new design led to numerous errors, operational difficulties, and failure to complete tasks. The scroll wheel and the color selection corresponded to design features that can become too salient and lead users to preferring a certain device. For that reason, our team ensures that design evaluations not only rely on the self-reported, subjective opinion of the users, but also on the unbiased, direct observation of their performance.

The paradox of preference versus performance described herein demonstrates the potential of design in affecting user preference, sometimes at the expense of the system’s usability. While interpreting the results of such testing, one must be cognizant not to bias his/her conclusion in favor of a design that in the end could be compromised.

### The Paradox of Choice: Less Is Often More

A number of studies have demonstrated how choice influences our buying decisions, selection of services, and ultimately how choice impacts our lives [17]. Choice consists of a mental decision-making process in which individuals have to judge merits among a range of options available and select one [18,19]. Although rooted in individual cognitive processes, extensive work over the years has been carried out in understanding how to influence choice by manipulating the access to information and how information is presented to individuals, with regard to marketing, interface design, and product design.

Although choice is often praised as being necessary for proper decision making, extreme situations can result in indecision and discomfort [17]. Schwartz [17] describes how excessive choice has impacted us as individuals and collectively as a society. Especially relevant here is his description of situations in which too much choice for individuals can potentially result in conditions in which a user makes poor choices, or no choice at all.

Within a health care perspective, designers can have the misconception that including more features in a product would be beneficial to caregivers and patients, who would now have a wider range of functionality and operational modes to use and more features to tailor their care. The pitfall is that, by including those additional features, one can lead caregivers to make poor choices, as described by Schwartz [17].

Health care is a highly demanding work environment where caregivers are generally under extreme pressure, which is a perfect situation for excessive choices to become overwhelming and a nuisance at a minimum, and safety hazard at its worst. Adding more choice and options to a single user interface can create uncertainty and distraction to the user. The complexity can create visual noise generated by the new features and cause users to be less efficient, make use errors, and generally provide them with a poorer user experience.

Within a health information technology domain, our teams have observed the effects of the paradox of choice consistently in the design and evaluation of electronic medical record (EMR) systems. To satisfy all possible end users from different specialties and different areas of a health care institution, designers include many features, functions, and information fields on a single user interface. EMR systems are renowned to overload users with choices on a single screen, creating a situation where users struggle to find the necessary information, function, or feature [20,21]. The consequence is that users now have to dig through numerous screens and tabs to find or enter the necessary information, leading to decreased performance, increased frustration, and unnecessary workload. EMR manufacturers have taken a one size-fits all solution that can severely impact the usability of the systems. Hence, EMR manufacturers must be aware of the paradox of choice to design future EMR systems that rely on simplified interfaces that present the user with a limited number of choices, facilitating access to information and reducing load on the user.

We need to be cognizant, however, that health care institutions fail to design their work environment for simplicity of workflow and standardization. Each health care institution prides itself for being unique. Consequently, manufacturers of health care technology have to navigate this complex environment and constantly make critical decisions: design a simple system to the specification of a few organizations or a complex system that fits most organizations? Nonetheless, engineers and designers must be aware of the *paradox of choice,* as during their effort to create a product that satisfies a greater audience, they may end up with an unusable product, which is often the case in EMR systems. The systemic issue with the lack of standardization must be addressed in the long run to ensure that medical technology can be properly designed to maximize benefits and mitigate usability issues. Health care must strive to harmonize their work environment and policies to increase standardization and consequently, facilitate the design of better technology.

**Figure 3:**
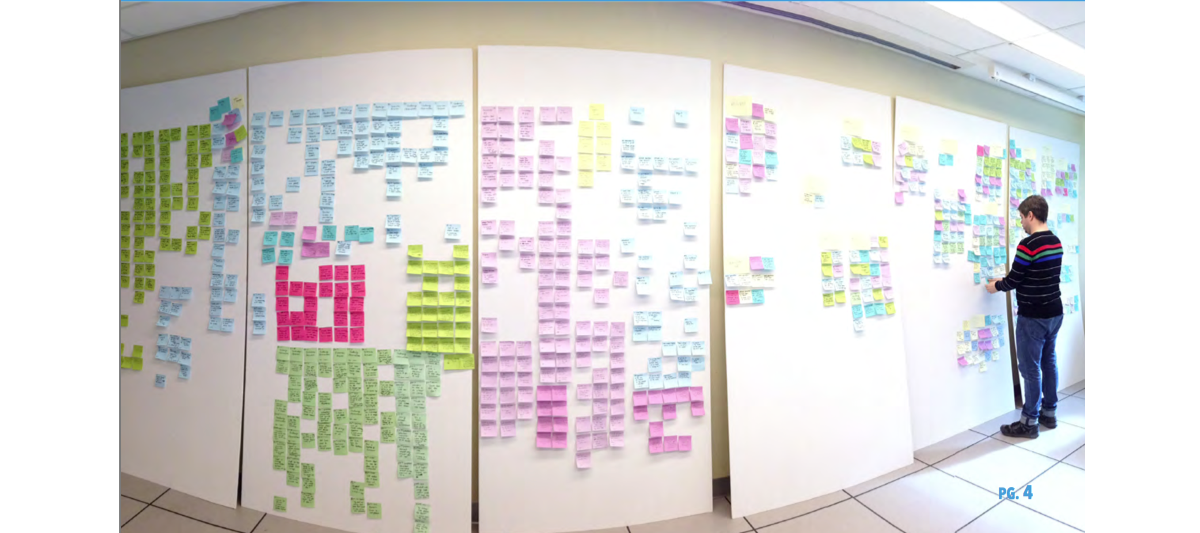
Usability labs at Toronto General Hospital, where we show the data analysis process through concept mapping and affinity diagrams.

This is not to suggest that only extremely simple systems with basic functionality are viable. A delicate balance needs to be cast where designers should aim for an interface in which users are not overloaded with excessive choices, while being inclusive enough to incorporate necessary features for proper operation of the system for the advanced user. Such systems can only be achieved through a detailed and careful design process that incorporates the needs and constraints of the final users (Figure 3).

## In the End, What Is Necessary for a Successful Design?

Usability testing and other HF methods are undoubtedly useful and powerful tools in the design process. However, one needs to be mindful of the pitfalls discussed here when designing systems and when evaluating the data collected through testing, as they may significantly influence the final design of a health technology. The paradoxes described in this article have the potential of skewing the understanding of the work domain and product requirements by presenting the designers with an incomplete and biased perception of the task. To design a product that is in alignment with the needs of its final users, designers must be aware of the paradoxes of expertise, preference versus performance, and choice, to ensure that their effect on product design is controlled or even mitigated.

**Figure 4:**
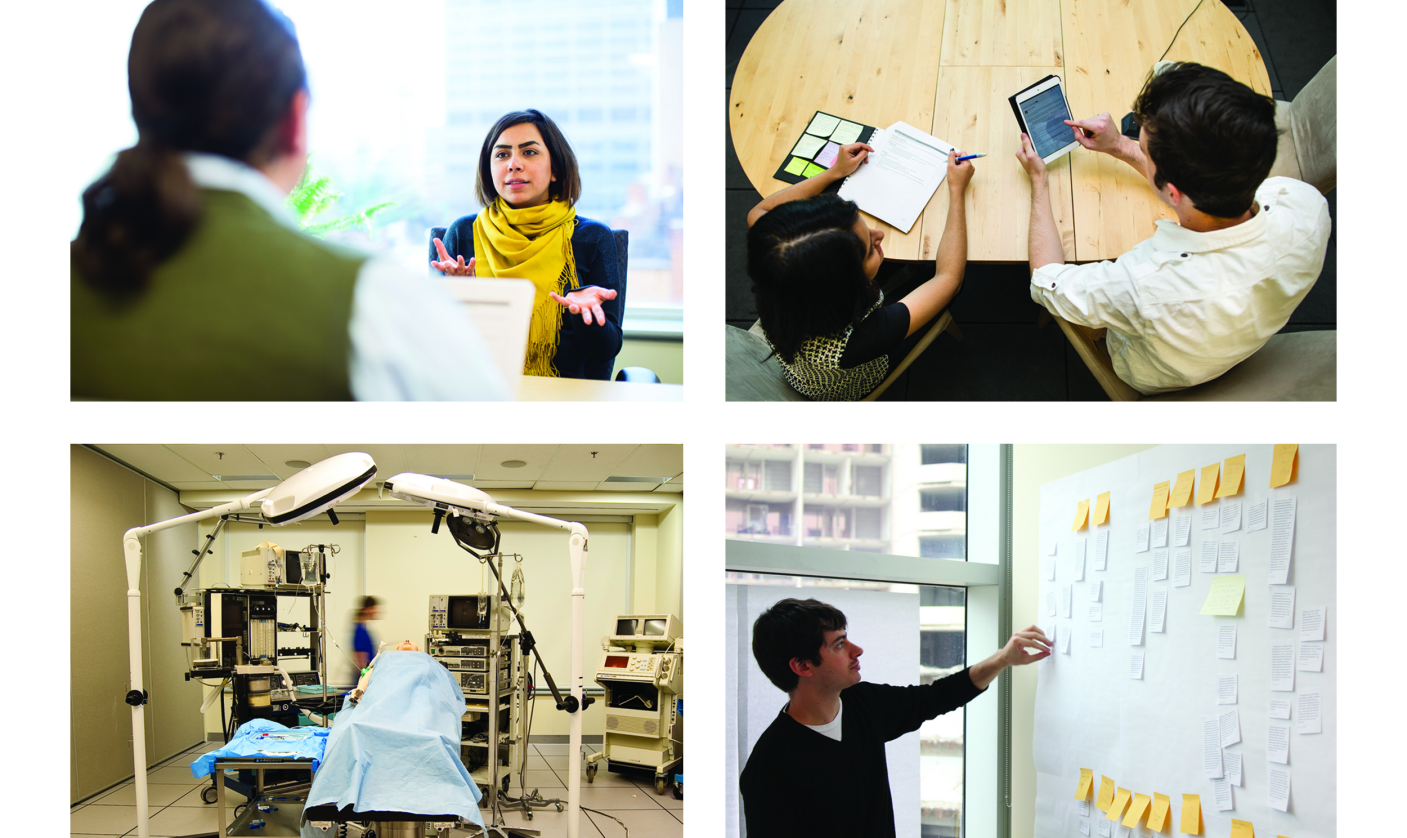
Examples of human factors methods used by human factors professionals at the University Health Network for designing and testing medical technology. Starting from the top left, clockwise, we showcase examples of interviews, cognitive walkthroughs, concept mapping, and usability testing.

The lesson to be learned from the paradoxes described in this paper is that to design health technology aligned with the needs of its final users, engineers and manufacturers must incorporate a gamut of UCD methods (Figure 4) in the design process to gain a comprehensive and realistic understanding of the work domain and user constraints. Observational methods such as cognitive walkthroughs and usability testing provide an opportunity to gather information about how users actually use the technology. The data gathered through these two methods can help minimize the impact of the *paradox of expertise* and the *paradox of preference versus performance*, allowing designers to focus on tailoring the technology based on unbiased usage data. Other methods such as interviews and concept mapping can be used to address the effects of the *paradox of choice*, creating opportunities for designers to identify the needs of each health care professional and organize the requirements into a manageable and tailored version of the technology.

A combination of methods is always necessary to ensure that the system being designed aligns with user needs and works toward bridging some of the gaps identified. Only then it is possible to focus on designing simple and tailored health technology that maximizes benefits to the users without overloading them with choice.

## Abbreviations

EMR: electronic medical record
HFP: human factors professional
UCD: user-centered design
